# Perspectives of trainees and examiners on communication skills assessment during online postgraduate psychiatry examination in Ireland

**DOI:** 10.1192/j.eurpsy.2022.2193

**Published:** 2022-09-01

**Authors:** M. Usman, G. Mccarthy

**Affiliations:** 1 St James’s Hospital Dublin, Liaison Psychiatry For Elderly, Dublin, Ireland; 2 NUI Galway, School Of Medicine, Galway, Ireland

**Keywords:** Objective Structured Clinical Examination, psychiatry, online, communication

## Abstract

**Introduction:**

Covid-19 pandemic caused a pivot to online clinical education and assessment across the globe.

**Objectives:**

To explore the views of psychiatric trainees and examiners on assessment of communication skills during online high stakes postgraduate examination.

**Methods:**

This study was designed as interpretive descriptive qualitative research. All candidates and examiners of the online Irish Basic Specialist Training exam in September and November 2020 were included. The respondents were interviewed by Zoom which were transcribed verbatim. Data was coded using NVivo20 pro and Braun and Clarke thematic analysis was used to draw various themes and subthemes.

**Results:**

A total of seven candidates and seven examiners from different training deaneries and specialties were interviewed with average duration of 29m 45s and 24m 20s respectively. The participants were largely satisfied with the online examination but did not consider it equal to face-to-face for picking nonverbal cues. The candidates were very conscious of eye contact while examiners placed more emphasis on overall professional behavior and patient engagement. All candidates preferred to continue online format post pandemic for practical reasons e.g., avoiding travel and overnight stay, while all examiners preferred to go back to in-person Objective Structured Clinical Examination due to some limitations in assessing physical and cognitive examination. However, continuation of online Clinical Formulation and Management Examination was agreed by both groups.

**Conclusions:**

The results of the study have shown different insights of two important stakeholders in a professional postgraduate psychiatry examination which can be useful to improve same exam and design similar assessments in other settings.

**Disclosure:**

No significant relationships.

